# Applications and methods utilizing the Simple Semantic Web Architecture and Protocol (SSWAP) for bioinformatics resource discovery and disparate data and service integration

**DOI:** 10.1186/1756-0381-3-3

**Published:** 2010-06-04

**Authors:** Rex T Nelson, Shulamit Avraham, Randy C Shoemaker, Gregory D May, Doreen Ware, Damian DG Gessler

**Affiliations:** 1 USDA-ARS, CICGR, 100 Osborne Dr. Rm. 1575, Ames, IA, 50011-1010 USA; 2 Cold Spring Harbor Laboratory, 1 Bungtown Road, Cold Spring Harbor, NY 11724, USA; 3 National Center for Genome Resources, 2935 Rodeo Park Drive East, Santa Fe, NM 87505, USA; 4 USDA-ARS, 1 Bungtown Road, Cold Spring Harbor, NY 11724, USA; 5 University of Arizona, 1657 E. Helen St., Tucson, AZ 85721, USA

## Abstract

**Background:**

Scientific data integration and computational service discovery are challenges for the bioinformatic community. This process is made more difficult by the separate and independent construction of biological databases, which makes the exchange of data between information resources difficult and labor intensive. A recently described semantic web protocol, the Simple Semantic Web Architecture and Protocol (SSWAP; pronounced "swap") offers the ability to describe data and services in a semantically meaningful way. We report how three major information resources (Gramene, SoyBase and the Legume Information System [LIS]) used SSWAP to semantically describe selected data and web services.

**Methods:**

We selected high-priority Quantitative Trait Locus (QTL), genomic mapping, trait, phenotypic, and sequence data and associated services such as BLAST for publication, data retrieval, and service invocation via semantic web services. Data and services were mapped to concepts and categories as implemented in legacy and *de novo *community ontologies. We used SSWAP to express these offerings in OWL Web Ontology Language (OWL), Resource Description Framework (RDF) and eXtensible Markup Language (XML) documents, which are appropriate for their semantic discovery and retrieval. We implemented SSWAP services to respond to web queries and return data. These services are registered with the SSWAP Discovery Server and are available for semantic discovery at http://sswap.info.

**Results:**

A total of ten services delivering QTL information from Gramene were created. From SoyBase, we created six services delivering information about soybean QTLs, and seven services delivering genetic locus information. For LIS we constructed three services, two of which allow the retrieval of DNA and RNA FASTA sequences with the third service providing nucleic acid sequence comparison capability (BLAST).

**Conclusions:**

The need for semantic integration technologies has preceded available solutions. We report the feasibility of mapping high priority data from local, independent, idiosyncratic data schemas to common shared concepts as implemented in web-accessible ontologies. These mappings are then amenable for use in semantic web services. Our implementation of approximately two dozen services means that biological data at three large information resources (Gramene, SoyBase, and LIS) is available for programmatic access, semantic searching, and enhanced interaction between the separate missions of these resources.

## Background

SoyBase[1] was originally developed as the USDA-ARS soybean genetics database. Since its inception, SoyBase has matured from a genetic map-based database to include the just-released soybean genomic sequence and its annotation. The SoyBase database contains numerous data types including QTL, locus and phenotypic data for soybean. Gramene [2,3]is a database for comparative genomics of the grasses, which offers a suite of tools and data for comparing different grass species. The Gramene database is a resource for comparative genetics and genomics of plants, which holds numerous classes of data related to the molecular biology, genetics and genomics of the species present in the database. The Legume Information System (LIS) [4] is a USDA-ARS funded information resource for comparative genetics across legume species. The mission of LIS is to help basic science researchers translate and leverage information from the data-rich model and crop legume plants to fill knowledge gaps across other legume species and to provide the ability to traverse interrelated data types. While SoyBase is a species-specific database for soybean (*Glycine max*, (L.) Merr.), it contains many of the same data classes as the Gramene database, including information on agronomically important plant phenotypes (traits) and genetically mapped quantitative traits, commonly referred to as QTLs (Quantitative Trait Loci). Soybeans are not grasses; they are legumes. Thus while SoyBase shares data types with Gramene, it shares data and evolutionary relevancy with LIS. SoyBase contains QTL and genetic information for soybean, Gramene includes QTLs identified for numerous agronomic traits in the grasses with information on associated traits and coordinates for their loci on various genetic maps, and LIS includes cross-legume comparative data. All three major information resources afford their users the ability to go to their respective websites and search or browse sequences, genes, traits and other data from major cereal crops (Gramene) or legumes (SoyBase, LIS), yet cross website scientific integration is laborious and largely unstructured.

Two challenges that SoyBase, Gramene, and LIS face today are discovery of relevant external web services and the integration of external data sources into integrated presentations for their users. To illustrate the discovery challenge from the perspective of a user, consider a researcher searching the web for QTL services, where--other than going to particular resources already known to the researcher such as SoyBase or Gramene--one has few discovery options other than beginning a search with web search engines such as Google. However, the results returned by Google when searching on the string 'QTL' or similar keys are varied in their context and relevancy. So instead of searching for web resources with the string 'QTL' with its lack of contextual relevancy, we seek a method to provide users with a way to find services that operate on formal QTL objects and other data types based on well defined data models. When we offer such contextualized services, they can be invoked directly by users with appropriate front-ends as well as integrated by us in our own informatic offerings. Additionally and importantly, analysis of our requirements shows that the term "users" needs to be interpreted broadly: we seek not just the capability to allow people to better find data and services, but to allow computers, without human assistance, to both discover and engage such resources. With this capability, SoyBase, Gramene, and LIS can deploy automated programs to discover, engage, assess, assimilate, and variously integrate disparate data and services to provide more productive resources for our users.

An immediate and well-known difficulty in discovering and engaging disparate data and services is the non-standard, idiosyncratic structure of data resources and the idiosyncratic ways common data is described. Under normal circumstances, a database administrator that wanted to transfer data between databases would first have to examine the external database schema, looking at the sometimes cryptic labels for the fields in each of the external database's tables, and try to determine if there is an analogous field in one's own database before populating data. This process would, of course, have to be repeated with each external database the administrator wished to integrate. Documentation helps direct this process, but utilizing that documentation as human readers is low-throughput and non-automated. Web service application programming interfaces (APIs) can alleviate some of the low level issues in data retrieval and transfer, but they do not standardize discovery and invocation across providers.

Major information resources such as NCBI eUtils [5] and EMBL-EBI [6,7] offer web services and to a lesser extent Gramene and Soybase with such services as Distributed Annotation Servers (DAS) [8]and the Genomic Diversity and Phenotype Connection servers (GDPC) [9] are moving in this direction. These interfaces allow programmatic search and retrieval of data and engagement of services. Furthermore, one can discover these services using specialized search engines such as BioCatalogue [10]. But a limitation of this approach is that the underlying technologies do not lend themselves easily to semantic markup [11,12]. Without semantic markup, it is virtually impossible to write generic programs to discover and engage services without low-throughput human intervention. Without infrastructural support, non-semantic services require programmers to write custom programs or scripts to engage and parse each individual web service--a process that inherently does not scale to thousands of resources on the web. Efforts to address this limitation exist. For example BioMoby [13] uses a classification scheme of data and service ontologies to allow web services and their data to be tagged using publicly available terms. Research into more formalized web service composition expands the domain into automated on-demand workflows [14,15] and Service-Oriented Computing [16]. Still, many of these approaches are built upon an *ad hoc *semantic that either does not lend itself to formalized reasoning, or is promising yet not sufficiently developed from research to production grade.

The issue of semantics and ontologies is non-trivial. It is long noted that it is difficult to agree on what to call something as (seemingly) simple as plant anatomy or genes. Terms may become overloaded, such as "locus" or "marker," yielding different meanings in different contexts. Ontologies and the discussions surrounding their creation have helped alleviate some of the ambiguity, or at least aided in the establishment common terms for the purpose of knowledge classification and data exchange. Yet the nature of classification itself raises substantial conceptual challenges beyond simple agreement on terms [17]. It is unclear if any static ontological approach can ever fully capture the rich diversity of concepts and instantiations seen in biology. Data schemas used by information resources will likely share major concepts, but it is equally as likely that specific implementations will also differ in ways that make integration of their contents laborious. We reject an approach where database designers would mold their web service offerings around a universal model, but rather we enable a model whereby there is a shared and vibrant semantic, building upon existing ontologies, and as appropriate, extending or creating new ontologies under a formal semantic.

To address this, we sought a system that is based on a simple, REST-based architecture (REpresentational State Transfer, [12]) using industry-standard semantic web--rather than web service--technologies. Semantic web technologies, such as the W3C-sanctioned language OWL, provide a formal semantic and logic for grounding web resource descriptions. We describe here our use of the Simple Semantic Web Architecture and Protocol (SSWAP) [18,19] to build a system that allows semantically robust description, discovery, and invocation of semantic web services. SSWAP affords data or service providers with the ability to describe their resources using semantics in a way that is both consistent with the use of shared community ontologies and amenable to formalized reasoning. This is particularly important when trying to transfer analogous data between distinct and independently created databases. SSWAP is a semantic web service architecture, protocol, and platform that allows users to re-use or create ontologies for their data, thus leveraging the efforts of many groups but still allowing web resources the ability to describe data that is unique to their offering or not addressed by other ontologies. SSWAP enables services to be described, discovered, and engaged based on the use of an extensible and formalize semantic, rather than the *ad hoc *conventions of simple lexical token matching.

## Methods

### RDF/XML and OWL-DL

SSWAP (Simple Semantic Web Architecture and Protocol) [18,19] uses standards as sanctioned by the W3C--the sanctioning body of the World Wide Web. SSWAP is a 100% OWL implementation. OWL (Web Ontology Language) [20] is the web standard for encoding a formal logic and specifically a first-order description logic in its variant OWL DL and newer OWL 2.0 dialects. OWL is fundamentally built upon RDF (Resource Description Framework) [21], RDFS (RDF Schema) [22], and XSD (XML Schema) [23]. SSWAP, as a lightweight specialization of OWL for semantic web services, achieves this by introducing exactly six classes, six object properties, and seven datatype properties [24,25]. For serialization, SSWAP uses the W3C recommendation of RDF/XML. We note for the reader that SSWAP's reliance on XML is solely as recommended by the W3C for messaging; functionally SSWAP neither depends on nor exploits XML syntactical or semantic side-effects.

### Ontologies

SSWAP recognizes that ontology content generation is best done by the community, for the community. Similarly, it recognizes the immense value in legacy work, even if those ontologies cannot be used for semantic web services in their current format. Thus SSWAP-compliant ontologies address the structural issues relevant to implementing a semantic web services system. Many legacy ontologies, such as those of the Open Biomedical Ontology (OBO) foundry [26] can be re-factored into semantic-web-service-compliant ontologies under automated services such as those available through the SSWAP web site [27]. A selection of these re-factored ontologies are hosted on the SSWAP ontology page [28]. Hosting at this web site is solely for community convenience: architecturally SSWAP ontologies may reside anywhere on the web.

SSWAP's use of ontologies is also aimed at addressing the social limitations often associated with ontologies and web services. How do we get agreement on the use of a term? SSWAP does not insist that all parties use the same term. Instead, SSWAP enables a marketplace of ontologies whereby anyone can put an ontology on the web for use in semantic web services. If such ontologies share nothing in common, then indeed there is no logical or semantic connection between terms. But in practice, ontology creation is laborious, and there is a strong incentive to reuse the work of others and extend it only when needed to address a local requirement. Thus SSWAP enables a "web of ontologies", where providers such as the OBO foundry and the Gene Ontology offer their ontological models, and users can mix, match, and extend terms as dictated by their specific requirements. Because all ontologies used in SSWAP are in OWL DL, the resultant admixtures are amenable to consistency checking and reasoning. There is no requirement for global consistency, thus locally created admixtures may be used ephemerally on a transaction-based model without breaking global behavior. SSWAP's use of OWL means that ontologies may be extended and used by third-parties under a formal semantic, without requiring explicit coordination.

Ontologies for each of the information resources of Soybase, Gramene, and LIS were constructed in consultation with each other to minimize work and identify shared concepts, but actual ontology creation was done independently at each site using the Protégé editor [29]. Where agreement was not found, ontologies were specialized to each resource using OWL constructs such as subsumption (subclassing). RDF/OWL files representing the ontologies for each database were also generated using the ontology editor Protégé. These documents were split into their component class and property terms using the SSWAP service "Split Owl Ontology Files" [27]. The resulting files were then hosted by each information resource.

### Resource Description Documents

**Figure 1:**
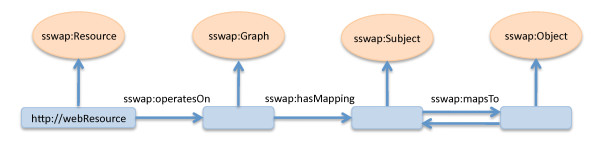
**The Canonical Graph**. SSWAP uses a canonical structure of relationships between a web resource (the blue http://webResource icon on the left) and its offering. Specifically, a resource states that it maps some data (*sswap:Subject*), such as a lookup key into a database, into return data (*sswap:Object*), such as DNA FASTA sequences. Multiple subject to object and inverse mappings are possible. Blue icons represent RDF "things"--either URLs or blank nodes, orange ovals represent OWL classes, and arrows represent OWL predicates. The class *sswap:Graph *allows for nested data structures. The full protocol supports an additional *sswap:Provider *class and other predicates not shown (see http://sswap.info/protocol.jsp).

Semantic web services use SSWAP to define themselves on the web in a simple document called a Resource Description Graph (RDG). An RDG is only superficially similar to the web service WSDL (Web Service Definition Language) [30] document in that it allows the resource to describe its offerings, but it otherwise differs substantially in both design and implementation. This is because WSDLs are not founded in a formal logic, but instead are a specification of how to encode (tag) information about a service for automated extraction. This means that while WSDLs provide a syntactical standard for how to organize and parse information, they offer no formalism on how to infer semantics (*e.g*., how to differentiate or equate terms such as *myOntology:DNASequence *from *yourOntology:DNASequence *or other tags). In SSWAP, RDG's are 100% OWL DL. Semantic web service definitions are self-describing logical statements of what the service is, and what it performs. The RDG structure is grounded on the reserved classes and predicates of SSWAP, establishing the protocol [24]. The protocol establishes a fundamental relation between any given service and its input and output data transformation. This fundamental relation is called the canonical graph (Figure 1). The canonical graph closely follows the conceptual model of RDF in its notion of subject -> predicate -> object. An RDG is a standardized manner, amenable to reasoning, for a service to describe that it maps some input to some output, or vice versa. RDGs tend to be short documents--often no more than a dozen or so lines of RDF/XML. Yet they can be informationally dense. Because each URI to a term is itself a link to an OWL DL document, extracting the closure of the RDG (*i.e*., dereferencing all URIs and embedding the resultant OWL RDF/XML) can be automated to expand the RDG into hundreds of statements. This closure can in turn be sent to a reasoner, whereby implied inferences are made explicit. This can result in thousands of statements. This process is available for inspection using the on-demand validation and publication tool at the SSWAP web site [31]. To prepare each resource offered by Soybase, Gramene, and LIS, RDGs were prepared according to the examples available at the SSWAP site [32].

### Discovery server and services registration

**Figure 2:**
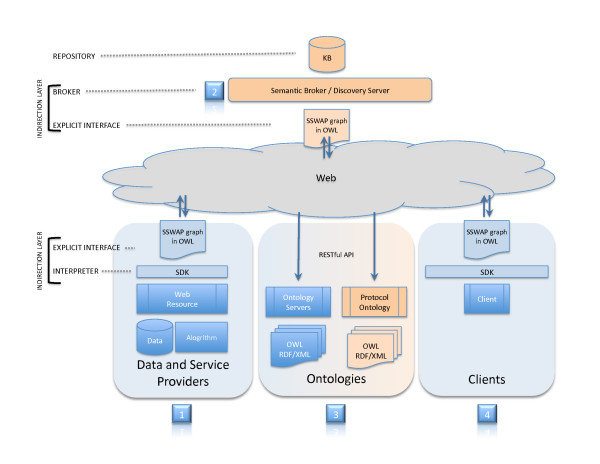
**A Resource Description Graph (RDG) Sample**. Actors--providers, clients, ontologies, and semantic search engines (Discovery Server)--meet in a semantic middle layer. The middle layer is instantiated as OWL documents, thereby enabling explicit descriptions amenable to formal reasoning. 1) Providers of data and services describe and publish their offerings; 2) these descriptions are available for semantic searching; 3) all actors use and reuse publicly available and extensible ontologies; 4) clients engage resources either directly or via a Discovery Server. Abbreviations: RDG: Resource Description Graph; SDK: Software Development Kit; KB: Knowledge Base.

Providers of data and services describe their offerings to the world by hosting RDGs on their web site (Figure 2). To allow their offerings to be discoverable by a semantic search engine, the site sends the RDG's URI to a third-party Discovery Server [33]. An open-source Discovery Server is hosted at http://sswap.info for this purpose. Upon receiving a publication notification, the Discovery Server executes a HTTP GET on the URI and reads in the content. If the content is a valid SSWAP graph, then the Discovery Server parses and reasons over the document and adds the content to its knowledge base for immediate on-demand semantic searching. If the document is not a valid graph, then the Discovery Server recognizes that the URI is not a SSWAP resource, and updates its knowledge base accordingly. Thus publication and deregistration are simply a matter of having the Discovery Server's internal model reflect the reality of the web. Architecturally, the model is amenable to spiders and web bots, whereby no active publication notification on the part of the provider is necessary, though we do not currently traverse the web for resources in this manner. Like virtually all systems on the web that use a snapshot of the web for backend processing, the model is subject to latency errors, whereby the Discovery Server's results are dependent on how it reflects the actual state of web resources at any given time. In this manner, the validity of the SoyBase, Gramene, and LIS service graphs were checked for format and consistency using the SSWAP "Validate Resources" service [31]. When each service's resource description graph (RDG) passed validation, it was registered with the Discovery Server and is available for semantic discovery and invocation.

### Search, discovery and activation of services

For browser-based, point-and-click interaction, clients may discover and engage services via the semantic search front-end at the SSWAP home page [19]. For non-browser, automated access, both discovery and invocation are fully integrated and supported in the SSWAP canonical graph model. To discover services programmatically (without the use of a browser), client programs send a graph to the Discovery Server, as exemplified with the example at the SSWAP web site [34]. Clients use the same publicly available web of ontologies as is available to providers to annotated their query graph called a Resource Query Graph (RQG). The Discovery Server returns a SSWAP graph with a mapping to all discovered resources. The actual discovery and matching process uses the power of OWL to perform semantic searching specifically relevant to semantic web services. Specifically, those services returned belong to a:

* subclass of the query graph's sswap:Resource, and

* superclass of the query graph's sswap:Subject, and

* subclass of the query graphs's sswap:Object.

Thus, discovered resources are exactly those semantic services which are guaranteed to be able to process and return the types of data requested by the client. It returns services that are as specific, or more specific, than the service class requested; (and) services that accept data types as general, or more general, than the input data type of the client; (and) returns data that is as specific, or more specific, than the return data sought by the client. For service invocation, the client sends the service a graph with the appropriate input data in the graph called a Resource Invocation Graph (RIG). Depending on the construction of the RIG, the data itself does not need to be serialized in the graph, but can be referenced indirectly by URLs. The client can also engage services directly without the Discovery Server, in a manner analogous to how anyone can use a search engine such as Google to find web sites or visit them directly.

SSWAP's knowledge base of statements underlying the Discovery Server is implemented in PostgreSQL 8.2. This is accessed by Hewlett-Packard's open source semantic middle layer, Jena [35], as a Java API to SPARQL [36], and augmented by a light-weight prototype Java API for SSWAP-specific coding. Jena plus the SSWAP API give the Java developer a package to manipulate SSWAP graphs in Java rather than in the lower level OWL or RDF.

## Results

### Gramene QTL ontology and semantic services

In order to describe the data available from Gramene, QTL, trait, and map ontologies were developed in OWL DL using the ontology editor Protégé. We also reused and extended other ontologies available at the SSWAP ontology portal [28]. Initially, the Gramene QTL ontology was constructed to define the data model based on the current data contained in the Gramene QTL class and those data types our services would provide. Thus we generated the ontology classes and their properties (*e.g*., 'name', 'symbol', 'synonym', etc.) based on the existing Gramene databases and their underlying query interfaces. At a later stage in collaboration with SoyBase, LIS, and SSWAP developers, we developed more general QTL, 'trait', 'map' and 'marker' ontologies. These ontologies are available via the SSWAP ontology portal [28]. The ontology terms that are specific for Gramene are available at http://sswap.gramene.org/vpin/ontologies/qtl.

Gramene acts as a service provider under the SSWAP semantic web services platform. Gramene currently offers 10 QTL resources allowing searching for QTLs by different means, such as by QTL metadata (accession ID, QTL symbol) as well as by trait (accession ID, symbol, synonyms, and category), species (common or scientific name), and linkage group (see Additional File 1). In order to describe these services 13 existing terms from four external ontologies were incorporated into the Gramene QTL ontology. These QTL services provide information such as traits, species, mapping information, location on a map, and other associated data. All of these services provide the same data and functionality offered via the Gramene main website, but are now formalized for semantic discovery and invocation.

These services can be discovered at the SSWAP discovery server [19]. Researchers may go to the Discovery Server at http://sswap.info, search for 'QTL' to discover Gramene's services as well as other resources that operate on formal QTL objects. These services may be invoked and data may be shared and integrated with other resources.

### SoyBase QTL and Locus ontology and semantic services

SoyBase also acts as a service provider under the SSWAP semantic web services platform. The return from the 'QTL' query described above will also contain a list of SoyBase services associated with QTLs. This is because an important data class in both the Gramene and SoyBase databases is the QTL class. In SoyBase, this class contains data derived from many published soybean trait mapping experiments. The database schema for this data class was systematically explored for semantic equivalence to concepts embodied in previously published ontologies at the SSWAP ontology site [28]. These equivalences were used to construct the shared ontological concepts describing the data classes across information resources. In cases where a clear equivalence with a published ontology was not evident, new terms were established as subclasses of the most applicable concept in the published ontologies. In a similar manner, predicates or properties used to tag string literals were introduced as needed.

In total, ontologies for two major data types in SoyBase have been created, one for the QTL class [37] and the other for the Locus class [38]. Thirty-four SoyBase-specific data type properties for the QTL class and 26 for the Locus class were created to fully describe each data class. A total of three external ontologies were used to reference terms from each of the classes. The construction of a data-type ontology was the first step in creating SSWAP services to allow discovery and access to the SoyBase data. In total six services for the QTL class and seven services for the Locus class were developed. A general description of each SoyBase QTL and Locus service is presented in Additional File 2. A more detailed explanation of each service can be found in the individual service's RDGs found at the SoyBase QTL and Locus URIs [39,40].

### LIS ontology and semantic services

LIS offers numerous variants on two basic semantic web services: 1) sequence retrieval (implemented via two complementary services); and 2) BLAST analyses (implemented as a single service for both DNA and RNA queries) (Additional File 3). Web front-ends to the services are at http://clovis.ncgr.org and can be discovered from either the top navigation bar of LIS (http://www.comparative-legumes.org choose Analysis -> Semantic Web Services) or the Discovery Server at http://sswap.info.

Sequences can be retrieved by one of four methods. Users can enter the Genbank accession number, the TIGR Transcript Assembly number, the TIGR Consensus number, or a marker symbol. In the case of the latter, marker symbols can be restricted to any combination of 21 species, though most of the marker symbols are from SoyBase and relevant for *Glycine max*. Upon execution, the service does not return the data *per se*, but returns a URL to the sequence in FASTA format. Over two million sequences are available for retrieval via this service. Users may also submit DNA or RNA sequences for BLAST against LIS maintained libraries. The underlying SSWAP semantic web service accepts parameterization of gapped alignments, expectation and extension thresholds, word size settings, and limits on maximum hits and returned alignments. Users can choose from any of 18 DNA library and 67 RNA library restrictions. The service returns a plain text BLAST report.

For both services, the same functionality wrapped for browser access at http://clovis.ncgr.org is available for non-browser invocation as a SSWAP semantic web service. Indeed, the web front-end simply wraps and engages the underlying semantic web service. This means that these services are discoverable by semantically-aware Discovery Servers and can be engaged remotely without human intervention.

### Semantic searching

Given the described semantic services, users can now search for QTL, trait, and mapping resources via interactive or programmatic engagement. Interactively, the Gramene, SoyBase, and LIS services are discoverable at http://sswap.info. The web front-end at sswap.info accepts uncontextualized simple text as input. It associates that text with both semantic tags and raw lexical metadata for semantic resources in the knowledge base. For example, searching on the word "taxonomy" matches to the property *taxonomyID *and the class *TaxonomyRecord *used by the "Gramene QTL information Retrieval for QTL Accession ID" service. This and other services are discovered. For any discovered resource, the user can click on the eyeglass icon on the results page and see a narrative on why any given resource was discovered.

Behind the web front-end is a semantic graph search back-end. This is also available at http://sswap.info/sswap/resources/queryForResources/inputForm.jsp. This back-end graph search allows resources to be discovered programmatically. A work-through example is provided for users at the web site.

## Discussion

Ontology identification or construction is an integral first step in the creation of these semantic web services. SSWAP's use of OWL means that providers can extend extant ontologies via a formalized subsumption semantic to connect their specialized requirements onto broadly accepted generic terms. This yields two distinct yet interconnected levels of ontology deployment:

1) First tier, generic ontologies, often previously published. These ontologies encompass the main concepts regarding such areas as sequences, taxonomy, and genetic map features;

2) Second tier, institutional ontologies. These are created via subclassing from the top level ontologies. These cover the institutional idiosyncratic requirements not covered by other published ontologies. To the degree that separate providers share these lower level requirements, groups may use these terms or subclass them too. This process incrementally builds a de facto community standard.

The ability to use subsumption semantics demonstrates some of the sociological aspects of SSWAP's architecture. Instead of having to develop a single ontology for each community in which all stakeholders have to agree, SSWAP allows developers to define classes or properties independently or in socially small groups, yet still under a formal semantic. Thus SSWAP allows the encapsulation of data present in any database into an RDF/XML OWL graph for data transport, without the necessity for alterations to the underlying database structure or programming and while enabling a mechanism for semantic integration. Since each ontology is by default published and accessible on the web, any actor can use the terms and concepts to describe their services as appropriate.

In all three cases presented here (SoyBase, Gramene, LIS), SSWAP service construction was largely dictated by existing database functionalities. However, there may be differences in what information is returned for a seemingly similar search. For example, SoyBase and Gramene each provide a service to look up data for QTLs. Both databases return QTL data, but the type of data returned from a simple QTL request varies dramatically (Additional Files 1 and 2, SoyBase QTL Report Service and Gramene QTL Information retrieval for QTL published symbol). The semantic relations of each service's input and output data is grounded by each terms' place in their respective ontologies.

In general, SoyBase and Gramene services were designed to respond to those requests deemed most common in terms of other sites' requesting data. Two types of requests are anticipated to be the most frequent: first, a specific request for a data item using its associated map symbol as a lookup key; and second, general requests for unspecified metadata associated with a data item. At this stage, key equivalence is determined by lexical matches on the key value. In the future, it will also be possible to determine equivalence by the use of controlled vocabularies such as the Gene Ontology (GO) [41] and Plant Ontology (PO) [42] when they have been incorporated into the SoyBase database schema.

As an example, the SoyBase services *QtlReportService *and *LocusReportService *take as input the map symbol for a soybean QTL or Locus and return a report back for the bulk of the data concerning the map symbol present in SoyBase. In order for other databases or researchers to find out what symbols are available, other services were created. The SoyBase *LocusTypeService *was created to deliver a list of all the "Types" of loci in the database. The *LocusByTypeService *was created to take in a locus type and return a list of all map symbols of that type. With this list one could then systematically retrieve all data from the locus class held in SoyBase. Similarly, the *QtlLGService *was created to take as input a soybean linkage group identifier and return a list of all QTL on that linkage group. This list could then be used to systematically retrieve data for each QTL.

Gramene offers several types of services, where each service allows searching for QTLs and associated information, based on a particular search. The *qtl-by-accession *and *qtl-by-symbol *services provide detailed information about specific QTLs. The information includes traits (accession ID, symbol, synonyms, category) from the OBO-style trait ontology (TO) hosted at Gramene, as well as species (common and scientific name), linkage group, map name, and location on a map (start and end position).

The so-called 'trait' services, namely, the *qtl-by-trait-accession*, *qtl-by-trait-symbol*, *qtl-by-trait-name*, and *qtl-by-trait-synonym *allow searching for QTLs related to a specific trait. Traits in Gramene are identified by the Trait Ontology [43] and are categorized according to trait categories, which are assigned by agronomic importance (anatomy, abiotic stress, biochemical, biotic stress, development, quality, sterility or fertility, vigor, yield). The *qtl-by-trait-category *service allows retrieval of QTLs associated to traits that belong to one of these trait categories.

The *qtl-by-species-common-name*, *qtl-by-species-scientific-name *and *qtl-by-linkage-group *retrieve a list of QTL accession IDs and symbols that belong at a particular species or linkage group. This information may then be used to retrieve further details about a particular QTL using the above mentioned *qtl-by-accession-id *or *qtl-by-symbol *services or any other semantic web service that provides information given a QTL accession ID and/or symbol.

The three LIS services (*getSequenceForIdentifier*, *getSequencesForMarkerSymbol*, and *blastSequences*) demonstrate a number of SSWAP architectural features: 1) they allowed for the development of a local, lightweight *SequenceServices *ontology to be built to address the lack of appropriate ontology terms for sequence comparison parameters for semantic web services, while also allowing it to be used in aggregation with the established Nucleic Acids Research categories [44,45] to classify both the services and their input and output data. This demonstrates SSWAP's ability to support ontology extension and aggregation; 2) the services accept both optional and required parameters for their invocation; 3) the services encapsulate varying degrees of complexity: the sequence retrieval service accepts four different types of input keys, while the BLAST service accepts both DNA and RNA sequences for comparison under thousands of different parameter and library combinations; 4) the services use URLs as indirection mechanisms to allow the return of arbitrarily large data sets. For the sequence retrieval service, a URL is returned that points to the FASTA sequence so that the data itself is not embedded in the return graph; 5) in the case of the *blastSequences *service, a legacy capability at LIS was simply wrapped to turn it into a new SSWAP-compliant service; 6) the SSWAP services were designed for programmatic access and analysis with LIS's more than two million sequences, but they are wrapped with web front-ends to provide point-and-click engagement; 7) the services are discoverable and evocable outside of their providers' web front-ends via discovery and invocation directly from the third-party Discovery Server [19].

Because of the relative lack of semantic web tools, much work was done in Protégé and simple text editors. This proved challenging at times because, as database administrators and bioinformaticians, we had to bridge into the world of ontologies, semantics, and reasoning. Additionally, OWL DL as a web-enabled, first-order description logic has both strengths and limitations. Some concepts such as subsumption (subclassing) are deeply veined within the language, thus offering both power and opportunities for abuse. In other cases, seemly simple requirements such as setting a specified number of required parameter values for a service are not easily modeled by OWL. The greatest benefit, though, probably comes to the end user. They are protected from these development issues and see only the relative easy way in which they can discover data and services or access web pages that themselves rely on the benefits of these underlying technologies.

## Conclusions

SSWAP provides a flexible method for semantically describing data and services. This is evidenced by the ability to accommodate varied but related interactions with three major yet independent information resources. The model provides the framework to semantically associate independent services regardless of the fact that the underlying data schemas share little in common.

Looking forward, cross-species comparisons are important for all three information resources (SoyBase, Gramene, LIS). Integration of controlled vocabulary terms is underway at Gramene and is now being implemented at SoyBase. When incorporation of Gene Ontology and Plant Ontology controlled vocabulary terms into SoyBase are complete, it will be possible to retrieve a list of loci, QTL or genes from either resource based on common GO (Gene Ontology) or PO (Plant Ontology) accession numbers. This will further facilitate cross-species genetic and genomic comparisons by providing another level of semantic equivalence between taxa.

## Abbreviations

LIS: Legume Information System; OWL: Web Ontology Language; PO: Plant Ontology; QTL:Quantitative Trait Loci; RDF: Resource Description Framework; RDG: Resource Description Graph; RIG: Resource Invocation Graph; RQG: Resource Query Graph; RRG: Resource Response Graph; SSWAP: Simple Semantic Web Architecture and Protocol; TO: Trait Ontology; WC3: World Wide Web Consortium; XML: eXtensible Markup Language.

## Competing interests

The authors declare that they have no competing interests.

## Authors' contributions

RTN carried out and prepared the SoyBase SSWAP ontologies and services and participated in the drafting of the manuscript. SA carried out and prepared the Gramene SSWAP ontologies and services and participated in the drafting of the manuscript. DDG created the SSWAP protocol and directed the research, participated in the design of the study and creation of the LIS services, and helped to draft the manuscript. RCS, DW, and GDM oversaw the implementation and conception of the SoyBase, Gramene, and LIS services respectively. All authors read and approved the final manuscript.

## Supplementary Material

Additional file 1 **Table 1**. Listing of semantic web services offered by the Gramene Database with their associated input and output data types. Click here for file

Additional file 2 **Table 2**. Listing of semantic web services offered by the SoyBase Database with their associated input and output data types. Click here for file

Additional file 3 **Table 3**. Listing of semantic web services offered by the LIS Database with their associated input and output data types. Click here for file
