# Supplementary material for: Applications and methods utilizing the Simple Semantic Web Architecture and Protocol (SSWAP) for bioinformatics resource discovery and disparate data and service integration
Source: BioData Min. 2010 Jun 4;3:3. doi: 10.1186/1756-0381-3-3 (PMC2894815; doi:10.1186/1756-0381-3-3)
Supplement: Additional file 1 — Table 1. Listing of semantic web services offered by the Gramene Database with their associated input and output data types. [file 1756-0381-3-3-S1.PDF]

**Table 1** Gramene Semantic Web Services.

| Service | Input Data | Output Data |
|---|---|---|
| *gramene:qtl-by-accession* | QTL accession ID | QTL accession ID<br>QTL symbol<br>Map name<br>Linkage group<br>Map start position<br>Map end position<br>Species scientific name<br>Species common name<br>Species taxonomy ID<br>Trait symbol<br>Trait name<br>Trait accession ID<br>Trait synonyms |
| *gramene:qtl-by-linkage-group* | Linkage Group ID | QTL accession ID<br>QTL symbol |
| *gramene:qtl-by-species-common-name* | Species common name | QTL accession ID<br>QTL symbol<br>Trait symbol<br>Trait name<br>Species scientific name<br>Map name<br>Map start position<br>Map end position |
| *gramene:qtl-by-species-scientific-name* | Species scientific name | QTL accession ID<br>QTL symbol<br>Trait symbol<br>Trait name<br>Species common name<br>Map name<br>Map start position<br>Map end position |
| *gramene:qtl-by-symbol* | QTL map symbol | QTL accession ID<br>QTL symbol<br>Map name<br>Linkage group<br>Map start position<br>Map end position<br>Species scientific name<br>Species common name<br>Species taxonomy ID<br>Trait symbol |

| | | |
|---|---|---|
| | | Trait name<br>Trait accession ID<br>Trait synonyms |
| *gramene:qtl-by-trait-accession* | Trait accession ID | QTL accession ID<br>QTL symbol<br>Trait symbol<br>Trait name<br>Species common name<br>Map name<br>Map start position<br>Map end position |
| *gramene:qtl-by-trait-category* | Trait category | QTL accession ID<br>QTL symbol<br>Trait symbol<br>Trait name<br>Species common name<br>Map name<br>Map start position<br>Map end position |
| *gramene:qtl-by-trait-name* | Trait name synonym | QTL accession ID<br>QTL symbol<br>Trait symbol<br>Trait name<br>Species common name<br>Map name<br>Map start position<br>Map end position |
| *gramene:qtl-by-trait-symbol* | Trait symbol | QTL accession ID<br>QTL symbol<br>Trait symbol<br>Trait name<br>Species common name<br>Map name<br>Map start position<br>Map end position |
| *gramene:qtl-by-trait-synonym* | Trait synonym | QTL accession ID<br>QTL symbol<br>Trait symbol<br>Trait name<br>Species common name<br>Map name<br>Map start position<br>Map end position |

*Input Data* and *Output Data* correspond to specific ontology classes and/or predicates. Abbreviations: *gramene*: http://sswap.gramene.org/vpin/. The semantic web service URL (the RDG) is a composite of the prefix and the service name; *e.g.*, *gramene:qtl-by-accession* is at http://sswap.gramene.org/vpin/qtl-by-accession. The RDG contains the URLs for all ontology terms used. For a human interface to the service, search for it at http://sswap.info or invoke it directly via the URL value of the *sswap:inputURI* property in the RDG.
