# Supplementary material for: Applications and methods utilizing the Simple Semantic Web Architecture and Protocol (SSWAP) for bioinformatics resource discovery and disparate data and service integration
Source: BioData Min. 2010 Jun 4;3:3. doi: 10.1186/1756-0381-3-3 (PMC2894815; doi:10.1186/1756-0381-3-3)
Supplement: Additional file 2 — Table 2. Listing of semantic web services offered by the SoyBase Database with their associated input and output data types. [file 1756-0381-3-3-S2.PDF]

**Table 2** SoyBase Semantic Web Services.

| Service | Input Data | Output Data |
|---|---|---|
| *soybaseL:soybaseLocusByTypeService* | Soybean locus type (SSR, RFLP, etc) | Locus map symbols |
| *soybaseL:soybaseLocusGermplasmService* | Locus map symbol | Germplasm names |
| *soybaseL:soybaseLocusMapService* | Locus map symbol | Genetic map names |
| *soybaseL:soybaseLocusQtlService* | Locus map symbol | QTL genetic map symbols |
| *soybaseL:soybaseLocusReportService* | Locus<br>gene<br>marker<br>map symbol | Locus alleles<br>Amplification information<br>Associated QTL map symbols<br>Locus comments<br>Locus enzyme homologs<br>Locus framework markers<br>Locus Genbank accession ID<br>Locus general remarks<br>Germplasms associated<br>Information providers<br>Genetic map name<br>Map start position<br>Map end position<br>Misc. protein homology<br>Nodulin homology<br>Locus synonyms<br>Locus gel pictures<br>Locus probe sequences<br>Restriction enzymes used<br>References<br>Related loci<br>Remarks<br>Locus sequence<br>SSR allele size<br>Locus type<br>Locus gene |
| *soybaseL:soybaseLocusSynonymService* | Locus map symbol | Locus synonyms |
| *soybaseL:soybaseLocusTypeService* | | Locus types |
| *soybaseQ:soybaseQtlLGService* | Linkage group name | QTL genetic map symbols |
| *soybaseQ:soybaseQtlMapPositionsService* | Linkage group name | QTL accession IDs<br>QTL map symbols |
| *soybaseQ:soybaseQtlMapRangeService* | Linkage group name | QTL genetic map symbols |

| | | |
|---|---|---|
| | QTL map symbols | |
| *soybaseQ:soybaseQtlMapService* | Linkage group name | QTL genetic map symbols<br>Map start position<br>Map end position |
| *soybaseQ:soybaseQtlPositionService* | Linkage group name | Linkage group<br>Map start position<br>Map end position |
| *soybaseQ:soybaseQtlReportService* | QTL map symbol | Analysis method<br>Candidate gene<br>Comment<br>Contains<br>Insect pests<br>Interacting loci<br>Locus association figure<br>Locus map symbol<br>QTL synonyms<br>Other QTL evaluated<br>Population type<br>Positive<br>Putative candidate genes<br>References<br>Number of plants bulked<br>Interval length<br>Interval R2<br>Intervals associated<br>Percent variation explained<br>R2 definition<br>Linkage group<br>Map start position<br>Map end position<br>Interval centroid<br>Related QTL<br>Parents<br>Pathology associated with QTL<br>Population high score<br>Population low score<br>TO accession ID<br>Trait name |

*Input Data* and *Output Data* correspond to specific ontology classes and/or predicates; see the service RDG (visit the service link) for reference to the specific ontologies used. Abbreviations: *soybaseL*: http://soybase.org:8080/sswap/locus/; *soybaseQ*:

http://soybase.org:8080/sswap/qtl/. The semantic web service URL (the RDG) is a composite of the prefix and the service name; *e.g.*, *soybaseL:soybaseLocusByTypeService* is at http://soybase.org:8080/sswap/locus/soybaseLocusByTypeService. The RDG contains the URLs for all ontology terms used. For a human interface to the service, search for it at http://sswap.info or invoke it directly via the URL value of the *sswap:inputURI* property in the RDG.
