# Supplementary material for: Applications and methods utilizing the Simple Semantic Web Architecture and Protocol (SSWAP) for bioinformatics resource discovery and disparate data and service integration
Source: BioData Min. 2010 Jun 4;3:3. doi: 10.1186/1756-0381-3-3 (PMC2894815; doi:10.1186/1756-0381-3-3)
Supplement: Additional file 3 — Table 3. Listing of semantic web services offered by the LIS Database with their associated input and output data types. [file 1756-0381-3-3-S3.PDF]

Table 3 LIS Semantic Web Services.

Service	Input Data	Output Data
lisI:getSequenceForIdentifier	*One of*:<br>Genbank Accession ID<br>Transcript Assembly Identifier<br>Transcript Consensus Identifier<br>Sequence ID	URL of data in FASTA format
lisM:getSequencesForMarkerSymbol	*Required*:<br>Marker symbol<br>*Optional*:<br>Taxon	URL of data in FASTA format
lisB:blastSequences	*Required*:<br>DNA FASTA sequence<br>or<br>RNA FASTA sequence<br>*Optional*:<br>Filter query flag<br>BLAST method<br>Display results format<br>Gapped alignments flag<br>Gap opening penalty<br>Gap extension penalty<br>Expectation threshold<br>Extension threshold<br>Word size<br>Maximum scores to report<br>Maximum number of alignments<br>Genomic or transcriptomic library	BLAST report

Input Data and *Output Data* correspond to specific ontology classes and/or predicates. Abbreviations: *lisI*: http://clovis.ncgr.org/sswap/resources/getSequenceForIdentifier/; *lisM*: http://clovis.ncgr.org/sswap/resources/getSequencesForMarkerSymbol/; *lisB*: http://clovis.ncgr.org/sswap/resources/blastSequences/. The semantic web service URL (the RDG) is a composite of the prefix and the service name; *e.g.*, *lisI:getSequenceForIdentifier* is at http://clovis.ncgr.org/sswap/resources/getSequenceForIdentifier/getSequenceForIdentifie r. The RDG contains the URLs for all ontology terms used. For a human interface to the

service, search for it at http://sswap.info or invoke it directly via the URL value of the *sswap:inputURI* property in the RDG.
